# Isocratic ion pair chromatography for estimation of novel combined inhalation therapy that blocks coronavirus replication in chronic asthmatic patients

**DOI:** 10.1038/s41598-023-27572-w

**Published:** 2023-01-06

**Authors:** Mohammed E. A. Hammouda, Yomna A. Salem, Saadia M. El-Ashry, Mohamed A. Abu El-Enin

**Affiliations:** 1 Department of Medicinal Chemistry, Faculty of Pharmacy, Mansoura University, Mansoura, 35516 Egypt; 2 Department of Pharmaceutical Chemistry, Faculty of Pharmacy, Horus University - Egypt, New Damietta, Egypt; 3 Department of Pharmaceutical Chemistry, Faculty of Pharmacy, Sinai University - Kantara Branch, Ismailia, 41636 Egypt; 4 Department of Pharmaceutical Chemistry, Faculty of Pharmacy, National University of Science and Technology, Nasiriyah, Iraq

**Keywords:** Analytical chemistry, Medicinal chemistry

## Abstract

A rapid and sensitive isocratic ion-pair chromatographic method was developed for the accurate analysis of ternary mixtures of formoterol, tiotropium, and ciclesonide in their novel combined inhalation that is widely used for the symptomatic treatment of patients with chronic obstructive disease. Analytical separation was performed using a C_8_ column and ion pair mobile phase composed of acetonitrile: acidified deionized water (55: 45% v/v) containing 0.025% sodium dodecyl sulfate. The pH was adjusted to 3.0 using orthophosphoric acid and eluted isocratically at 2.0 mL/min and 40 °C applying UV detection at 237 nm. The calibration ranges were found to be 0.3–9.0 µg/mL for formoterol, 0.45–13.5 µg/mL for tiotropium, and 10.0–300.0 µg/mL concerning ciclesonide. The proposed method exhibited good repeatability, accuracy, and sensitivity (R.S.D. < 2.0%). The approach is rapid (run time does not exceed 15 min) and achieves satisfactory resolution (resolution factors = 7.45 and 5.3 between formoterol and tiotropium and tiotropium and ciclesonide respectively). The sensitivity and the efficiency of the proposed method permit their successful estimation with a recovery percentage ± SD of 99.33% ± 0.43 for formoterol, 99.15% ± 0.60 for tiotropium, and 99.90% ± 0.41 for ciclesonide.

## Introduction

Formoterol Fumarate (FF), (E)-but-2-enedioic acid;N-[2-hydroxy-5-[(1S)-1-hydroxy-2-[[(2S)-1-(4-methoxyphenyl)propan-2-yl]amino]ethyl]phenyl]formamid, is a long-acting β_2_-adrenoceptor agonist^1^. It is used to treat asthma and chronic obstructive pulmonary disease (COPD) by relaxing smooth muscles^1^. Tiotropium bromide (TIO), (1α, 2β, 4 β, 5α,7β)-7-[(Hydroxydi-2-thienylacetyl)oxy]-9,9-dimethyl-3-oxa-9-azoniatricyclo [3.3.1.02,4] nonane bromide, is a long-acting muscarinic antagonist^1^. It decreases the contractile tone of smooth muscle, and mucus production, providing a bronchodilator effect in asthmatic patients and complicated therapy for COPD^1^. Ciclesonide (CIC), pregna-1,4-diene-3,20-dione, 16,17-[[(R)cyclohexylmethylene] bis(oxy)]-11-hydroxy-21-(2-methyl-1-oxopropoxy), (11β, 16α), is an anti-inflammatory steroid^1^. It is mainly administered by inhalation for the treatment of asthma and allergic rhinitis^1^.

A review of the literature revealed that various methods for determining FF have been published, including spectrophotometry^2,3^, gas chromatography (GC)^4^, RP-HPLC^5,6^, and liquid chromatography-tandem mass spectrometric method (LC–MS–MS)^7^, ultra-performance liquid chromatography-tandem mass spectrometric method (UPLC–MS–MS)^8^, and capillary electrophoresis^9^. Other techniques for determining TIO have been reported, such as spectrofluorimetry^10^, GC^11^, HPTLC^12^, HPLC^13,14^, HPLC–MS^15^, and HPLC–MS-MS^16^. Besides, the following methods were reported for CIC determination including spectrophotometry^17^, RP-HPLC^18–20^, and LC–MS–MS^21^.

Triohale inhaler is a combined medication including FF; long-acting β_2_-agonist (LABA)/TIO; long-acting muscarinic M_3_ antagonist (LAMA)/CIC; inhalation corticosteroid (ICS) used for long-term management of severe COPD^22,23^.

By targeting non-structural protein 15 (NSP15), an endoribonuclease that helps evade host detection of viral double-stranded RNA, the corticosteroid ciclesonide as an effective inhaled corticosteroid (ICS) appears to reduce the ability of severe acute respiratory syndrome-coronavirus-2 (SARS-CoV-2) to replicate in vitro^24^. SARS-CoV-2 infection can be prevented by ICS; ACE2 gene expression is much lower in the sputum of COPD and asthma patients who use ICS relative to those who do not^25,26^. Furthermore, studies in mice have shown that ICS inhibits type 1IFN development, which reduces ACE2 expression^25^. Although the suppression of type 1IFN secretion may decrease the host defense, the associated reduction of ACE2 expression could help in the protection against SARS-CoV-2 cellular entry. Moreover, the effect of inhaled budesonide with other medication in the regulation of interleukin-19 (IL-19) levels in the saliva of patients with COVID-19 was recently studied^27^. These findings suggest that ICS, especially ciclesonide, could protect COPD patients from COVID-19 and that it may be a promising drug candidate for further development^28^.

The determination of FF, TIO, and CIC in their combined dosage form has been attempted using only a few techniques, including HPLC^29–31^. However, the chromatographic methods available for the simultaneous determination of FF, TIO, and CIC in their mixture have lower sensitivity and linearity ranges than the proposed IPC method. Compared to the reference HPLC method^31^, the developed method applies an isocratic run to separate FF, TIO, and CIC simply without performing an exhausting gradient run, in addition, it consumes a lower percent organic modifier compared to the reference methods that consume 90 mL, the proposed method is much rapid, where the run time that does not exceeds 15 min compared to the reference method that reaches 22.0 min^31^.

The addition of ionic organic compounds to the mobile process promotes the creation of ion pairs with charged analytes in ion-pair chromatography (IPC). Ion pair reagents (IPRs) will enhance the elution times of the charged substances selectively in the reversed-phase mode. IPRs form a non-charged complex by ion-pairing with the charged polar analyte, before the adsorption to the stationary surface, which was proven with NMR studies^32^. Moreover, the equilibria of the IPRs can be disturbed at larger injection volumes injected generating severe analyte peak distortions^33,34^. Ion pair chromatography has many advantages over ion-exchange chromatography, including ease of buffer preparation, a broad range of carbon chain lengths for improved retention and separation, substantially reduced separation time, simultaneous separation of non-ionized and ionized analytes, highly reproducible performance, and improved peak shape^35–43^.

The current research aimed to develop a precise isocratic IPC method for separating CIC in the presence of FF and TIO in raw materials, synthetic mixtures, and metered dose inhalers. To maximize the separation and quantitation of the studied drugs, various experimental parameters were carefully considered.

## Materials and methods

### Chemicals

Analytical reagent grades for all the applied chemicals and HPLC grade for applied solvents were utilized throughout the analysis.

Formoterol fumarate (99.92% purity), tiotropium bromide (99.80% purity), and ciclesonide (99.40% purity) were friendly supplied by sigma pharmaceutical industries company in Steinheim, Germany. They were analyzed as delivered without further purification.

HPLC-grade methanol, acetonitrile, and sodium dodecyl sulphate were purchased from Fischer chemicals in Germany. Orthophosphoric acid was purchased from El-Nasr pharmaceutical company in Egypt.

Triohale Inhaler; (Label claim: 6.0 μg Formoterol fumarate, 9.0 μg tiotropium bromide, and 200.0 μg ciclesonide /buff) (delivered dose), manufactured by Cipla was obtained from commercial sources.

### Apparatus

Separations were conducted by applying a Perkin Elmer-TM Series 200 chromatograph provided with a 20 μL loop Rheodyne injector valve and a 237 nm UV/VIS detection wavelength. For data collection and processing, the Complete Chrom Workstation (Massachusetts, USA) has been implemented. Using the Merck solvent L-7612 degasser and the Millipore filter (Sibata), the mobile stage was filtered and degassed, respectively. A Consort NV P-901 pH-Meter (Belgium) for measuring the pH.

### Columns and mobile phases

CLC Shim-Pack C_8_ column (250 mm, 4.6 mm, 5.0 µm, Shimazu—USA) was utilized for the separation of the provided mixture at 40 °C. The mobile phase consisted of 55% v/v acetonitrile: 45% v/v deionized acidified water containing 0.025% sodium dodecyl sulphate (SDS) adjusted to pH 3.0 using a diluted Orthophosphoric acid (OPA) solution. These components were equivalently combined in an ultrasonic instrument for half an hour, membrane filtered through a filtration unit of 0.45 µm made in Ireland, and an ultrasonic unit utilized to remove gases consuming a time of ten minutes. The flow rate was pushed at 2.0 mL/min with UV measurement at 237 nm. The mobile phase stability was preserved for two weeks.

### Standard solution preparation and procedures

Twenty-five mg of FF, TIO, and CIC were added to a separate 50 mL measuring flask enclosing methanol solvent, to get 500 μg/mL stock solutions of FF, TIO, and CIC. These prepared aqueous solutions were additionally diluted with methanol to get working solution concentrations within the prerequisite concentration range. The stock solutions were kept cold in the refrigerator at 4 °C, protected from light.

#### General procedures and calibration curves

The concentration ranges of 0.3–9.0, 0.45–13.5, and 10.0–300.0 µg/mL of FF, TIO, and CIC, respectively, were achieved using the proposed chromatographic method by transferring specific amounts of FF, TIO, and CIC working solutions into 10 mL volumetric flasks. Additional dilution of the flasks was performed utilizing the mobile phase prior to the injection into the chromatographic system. 20 μL was the volume of injection of aliquots and the separation was achieved at 40 °C. The curves of calibration were drawn by applying peak area measured values against the final adjusted concentration for the examined drug, as a result, regression line equations were then estimated.

#### Determination of the FF, TIO, and CIC in the synthetic mixtures

Measured aliquots of working FF, TIO, and CIC solutions preserving the pharmaceutical proportion of 1: 1.5: 33.3 were transferred into a series of 10 mL volumetric flasks. The steps were carried out as mentioned under “Development of calibration curves” Calculating the percent recoveries utilizing the provided regression equations.

#### Determination of the FF, TIO, and CIC in their combined metered-dose inhaler.

Ten actuation doses of Triohale MDI were brought via vacuum into separate volumetric flasks of 10 mL containing methanol to provide 6.0 µg/mL of FF, 9.0 µg/mL of TIO, and 200.0 µg/mL of CIC (according to their pharmaceutical ratio). Additional dilution of the flask was done by mobile phase to give solutions that have a concentration of 0.6 µg/mL of FF, 0.9 µg/mL of TIO, and 20 µg/mL of CIC. Species filtrations were applied through 0.45-µm membrane filters before the sample’s injection into the chromatographic system. A similar method expressed beneath “Development of calibration curves” was carried out to determine three separate sample concentrations chosen precisely within the concentration operational range for each medication in their inhaler preparation. The nominal percent of the pharmaceutical formulation was estimated using the appropriate regression equation.

## Results

### IPC method

**Figure 1:**
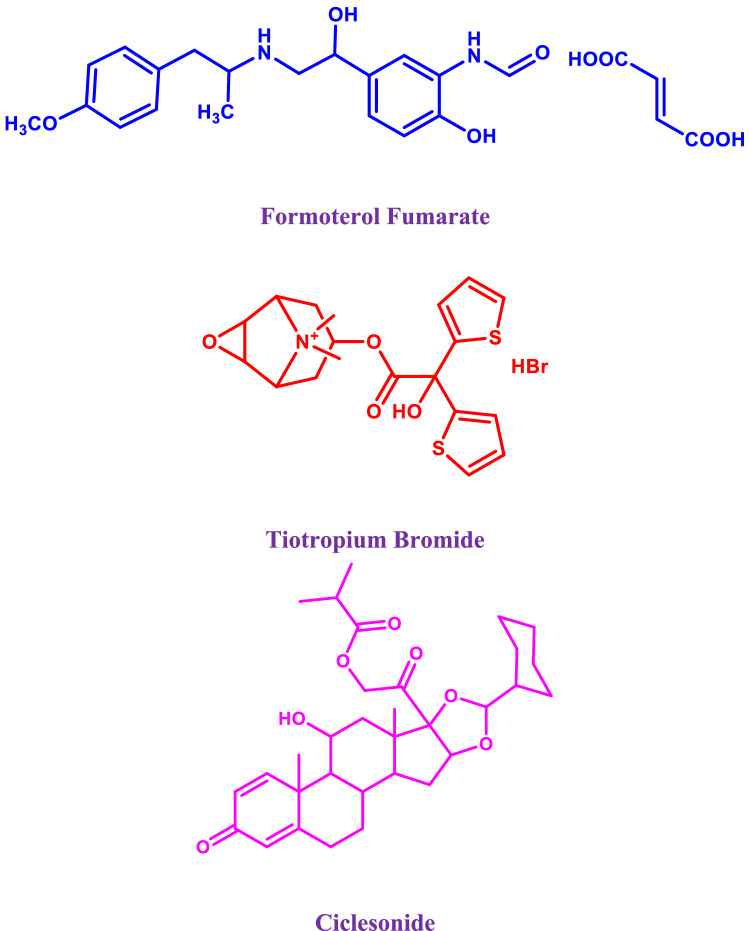
Structural formula of formoterol fumarate (FF), tiotropium bromide (TIO), Ciclesonide (CIC).

**Figure 2:**
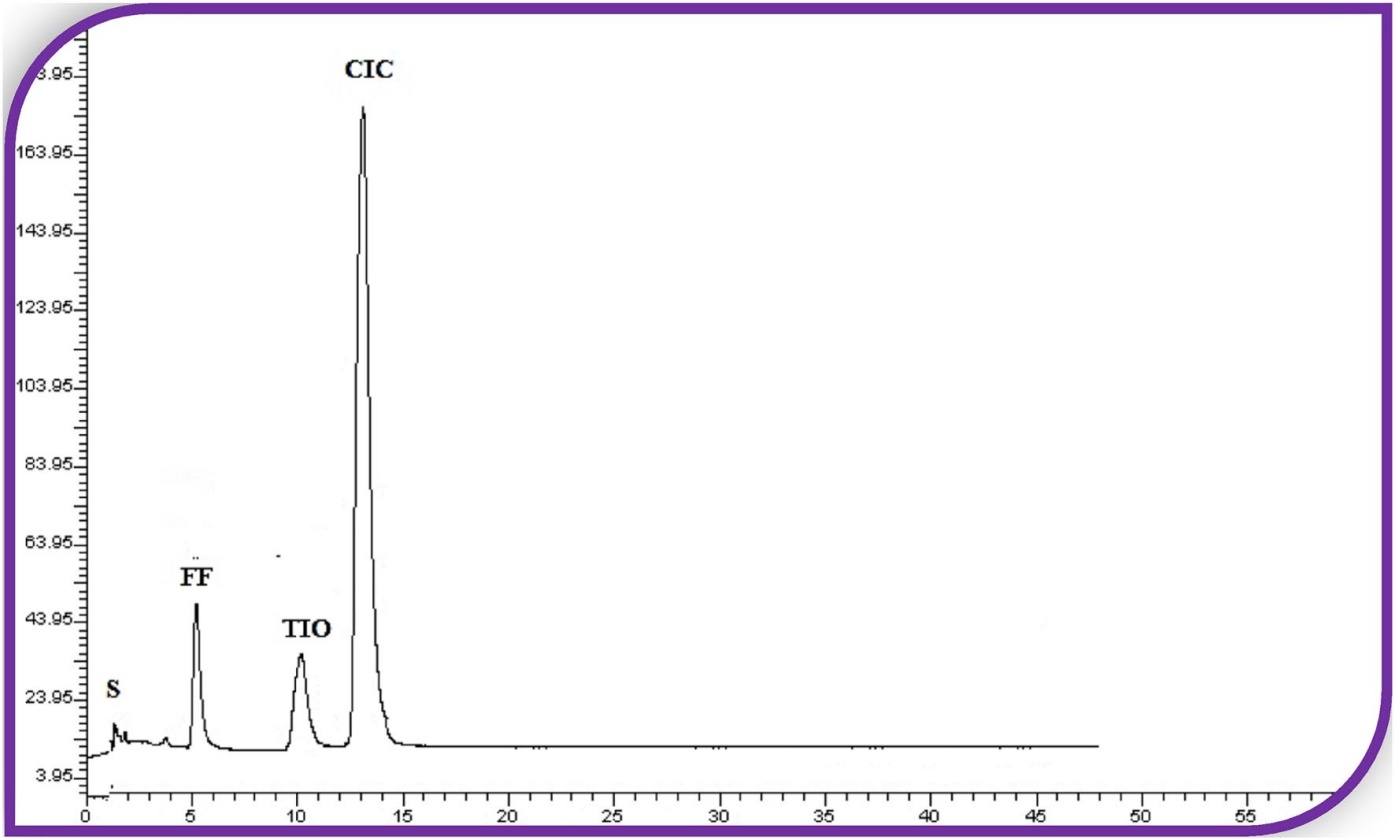
Typical chromatogram for the separation of FF (2.4 µg/mL, 5.8 min), TIO (3.6 µg /mL, 10.5 min.) and CIC (80.0 μg/mL, 13.3 min) in their synthetic mixture using ion pair mobile phase of the specified optimum characteristics.

The provided IPC method provides efficient separation between formoterol fumarate, tiotropium bromide, and ciclesonide (Fig. 1) with satisfactory resolution in a reasonable elution time. The achieved chromatogram for the studied mixture of FF, TIO, and CIC solution containing 2.4, 3.6, and 80.0 µg/mL and 0.6, 0.9, and 20.0 µg/mL in the synthetic and metered-dose inhaler, respectively are represented in Figs. 2 and S1, respectively. All the chromatographic parameters were also considered and optimized (Table 1).

**Table 1:** Optimization of the chromatographic parameters for separation of the FF, TIO and CIC by the proposed IPC method.

Resolution (Rs)		No. of theoretical plates(N)			Parameter	
		FF	TIO	CIC	Rs_1_	Rs_2_
pH	2.5	1590	1060	3680	7.20	4.65
	3.0	2020	1530	3910	7.45	5.3
	4.0	1630	1420	3500	7.09	4.2
	5.0	900	1504	2486	6.85	4.5
Ratio of [Acetonitrile: Acidified water] containing 0.025% SDS	(65:35)	Overlap	1600	Overlap	–	3.2
	(60:40)	2500	Overlap		4.23	–
	(55:45)	2020	1530	3910	7.45	5.3
	(50:50)	1010	902	1810	7.14	4.15
Conc. of SDS (%)	0.015	1670	1020	2500	6.81	4.2
	0.02	1868	1355	3100	6.96	4.96
	0.025	2020	1530	3910	7.45	5.3
	0.03	1752	1509	3663	7.3	4.75
Effect of column temperature	25 °C	834	1011	1974	6.2	3.47
	35 °C	1905	1480	3090	7.33	5.01
	40 °C	2020	1530	3910	7.45	5.3
	45 °C	1810.9	1320	2500	7.01	4.3
Flow rate (mL/min)	0.5	602	502	1702	6.01	3.5
	1.0	1050	920	2720	6.82	4.1
	1.5	1520	1220	3010	6.95	4.2
	2.0	2020	1530	3910	7.45	5.3

Where: Resolution (R) = 2 ∆ t_R_/W_1_ + W_2_. Number of theoretical plates (N) = 5.45(t_R_/ W_h/2_)^2^. Rs_1_ between FF and TIO and Rs_2_ between TIO and CIC. t_R_ is the retention time of the substance measured from the point of injection and t_M_ is the retention time of a non-retained marker. W_h/2_ is the peak width at the half height. W_1_ and W_2_ are the width of the peaks of the two components at their bases.

## Discussion

FF, TIO, and CIC are combined in their novel MDI to improve the treatment of asthma and COPD in patients, recent research suggests that ICS, especially ciclesonide, could protect COPD patients from COVID-19. To date, no isocratic IPC method has been described for the separation of this challenging ternary mixture ratio (1: 1.5: 33.3) for FF, TIO, and CIC, respectively. The developed method provides an isocratic run, higher sensitivity, and lower elution time that does not exceed 15 min, compared to the previously compared method which reaches 22 min^31^.

FF, TIO, and CIC are combined in the metered-dose inhaler to enhance the treatment of COPD. They have distinct lipophilicity that is challenging to resolve them in the same chromatographic run. CIC is more lipophilic than FF, and TIO as provided by log *p* values (log *p* values are 1.9, − 4.4, and 5.3, respectively)^29^.

IPC is a frequently used approach for resolving polar and non-ionic sample mixtures in a single run. This approach enhances peak symmetry and reduces separation time while maintaining high results and reproducibility. The key cause of band broadening of a porous C_18_ column stationary phase is the mass transfer, which could be minimized by utilizing smaller particles (3.5 μm), a shorter column (100 mm), decreasing the flow rate, or adding an ion pair reagent like SDS to the running mobile phase. The ion-pair chromatographic approach was used to quantify the FF, TIO, and CIC in raw materials as well as in combined dosage form.

Different results were obtained without the use of the ion-pairing agent SDS. The use of a 45:55 percent V/V mixture of acidified deionized water and acetonitrile resulted in an un-retained peak for TIO. CIC was delayed on the column for more than 20 min after acetonitrile was replaced with methanol, resulting in a broad peak for FF and CIC. In a suitable chromatographic elution period, well-defined resolved peaks were developed by adding 0.025 percent SDS as an ion pair reagent to a mixture of acetonitrile and acidified deionized water mobile phase (55:45% v/v), as shown in Fig. 2. Thus, in a short chromatographic run time, this approach efficiently separates the FF, TIO (Rs_1_ = 7.45), and TIO, CIC (Rs_2_ = 5.3).

To improve sample separation of basic drugs, the ion-pairing agents such as alkanesulfonates utilized must carry a negative charge. SDS is considered a successful ion-pairing reagent because it is inexpensive and readily available, and it has the opposite charge to the basic analytes (FF and TIO) that carry a positive charge under the separation conditions. For the separation of these bulky analytes (FF, TIO, and CIC), the bulkiness of the SDS ion pair is favored over other ion pairs with shorter chain lengths. According to the ion-pair formation theory, the charged part of SDS interacts with the analyte, while the hydrophobic part interacts with the stationary phase where the ion-pairing reagent and the selected sample form none charged paired complex. As a result, the sample can be retained and distributed between both phases within a reasonable run time of less than 15 min (Figs. 2, S1)^35,36^.

### Method development

The most relevant chromatographic factors influencing the separation of the mixture tested were carefully examined and analyzed. In terms of the number of theoretical plates and the resolution, the USP guidelines^44^ specify the chromatographic performance measurement and the results are presented in Table 1.

#### The Stationary phase

Performance of three columns were checked, utilizing columns including the CLC Shim-Pack C_8_ column (250 mm, 4.6 mm, 5 µm particle size, Shimazu—USA), C_18_ column (150 mm, 4.6 mm, 5 µm particle size, Knaur—Munich, Germany), and CN column (100 mm × 3.6 mm, 3 µm particle size, Knaur—Munich, Germany). The tested procedure approved that, a good, calculated resolution with symmetrical peaks was achieved after utilizing the C_8_ column. Neither the C_18_ column (150 mm length) can separate hydrophilic TIO in presence of highly lipophilic CIC in the same run nor CN column (100 mm length) provides well-resolved peaks.

#### The mobile phase

##### Choice of detection wavelength

To resolve the studied mixture, separate wavelengths over the range of (200–260 nm) were examined. The most appropriate wavelength for UV estimation was 237 nm because of high detection limits for the combined medications, especially for TIO which has low UV readings compared to FF and CIC at their prepared concentrations to satisfy linearity pharmaceutical ranges, allowing their simultaneous estimation in inhaler dosage form applying their recommended medicinal ratio.

##### The effect of pH

**Figure 3:**
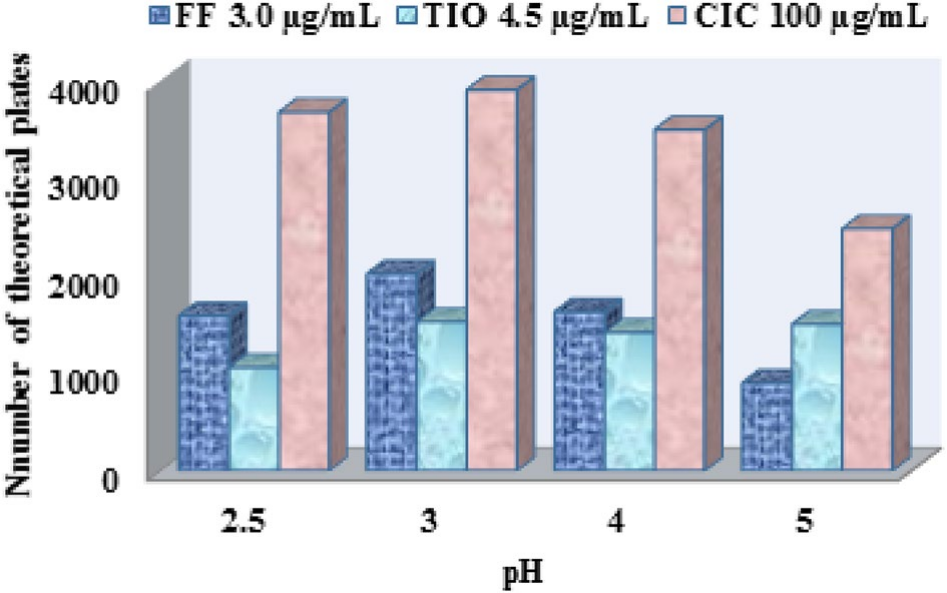
pH effect on the number of theoretical plates of FF 3.0 µg/mL, TIO 4.5 µg/mL and CIC 100.0 µg/mL using mobile phase consisting of acetonitrile: acidified deionized water containing 0.025% SDS (55: 45% v/v). Flow rate, 2.0 mL/min, column temperature 40 °C and UV detection at 237 nm.

Using an increasing amount of orthophosphoric acid, the mobile phase's pH was modified over the 2.5–6.0 range. The variation in the hydrophobicity and ionization constants of the drugs was presented by their log *P* (octanol/water) and pK-a values, respectively. FF has a log *P* value of 1.9 and two pK-a values of 8.6 and 9.8 (DrugBank accession number DB00983) due to the presence of a secondary amino group and phenolic OH group, respectively. TIO has a log *P* value of − 1.8 and pK-a value of 10.4 (DrugBank accession number DB01409), while CIC has a log *P* value of 4.08 and pK-a values of 14.7 (DrugBank accession number DB01410). There was no significant difference in the elution times of FF and TIO when the pH was raised to 5.0 owing to their basic nature within the studied pH range, so they will be completely ionized and carry a positive charge that pairs with the negatively charged part of SDS as pairing reagent and eluted first. As seen in Table 1, the pH value of 3.0 was selected for separation and determining the quantity of the analyzed mixture since it efficiently resolves drug peaks FF, TIO (R_s_1_ = 7.45) and TIO, CIC (R_s_2_ = 5.3) in an acceptable chromatographic run (< 15 min.) while also ensuring optimal efficiency of the resolved peaks as revealed by the number of theoretical plates (N) (Fig. 3).

##### The ratio of mobile phase components

The percent ratio of mobile phase components affects the retention time and resolution of this ternary mixture. The retention time of the CIC was sharply reduced to 5.9 min using the ratio of ACN to water (65:35, v/v), causing overlapped FF and CIC peaks, and the retention time of the CIC was reduced to 10.2 min using the ratio of acetonitrile: water (60:40, v/v), causing overlapped TIO and CIC peaks. The retention time of the tested compound was significantly increased (> 22 min.) once the ratio of acetonitrile to water (50:50, v/v) was applied, with a subsequent reduction in the separation efficiency as revealed by a sharp decrease in N value. (Table 1). The optimal ratio found to be (55:45, v/v) acetonitrile: deionized water has an SDS concentration of 0.025% and pH adjusted to 3.0. This developing system gives high detection limits, and satisfactory resolution in addition to optimum efficacy of the resolved peaks as shown by the maximum number of theoretical plates (N) (Fig. S2).

##### Concentration of SDS

The different SDS concentrations in the range of (0.015–0.03%) were tested in the mobile phase composed of 45% deionized water (pH 3) and 55% ACN, as shown in Table 1. As shown in Fig. S3, it was observed that decreasing SDS concentration drops the proficiency of the studied drug mixture as shown by lower values of N and a decrease in resolution between FF, TIO (R_s_1_ = 6.81) and TIO, CIC (R_s_2_ = 4.2). Meanwhile**,** raising the concentration of SDS to 0.03% exhibits a slight decrease in N. The optimum SDS concentration was 0.025% concentration as it provides optimum resolution, best linearity, reasonable retention time, and effective separation (Fig. S3).

##### Effect of the column temperature

The effect of column temperature on the resolution of the studied drugs was examined in a range of 25–45 °C. When the column temperature was maintained at 25 °C, the chromatographic parameters including N and R_s decreased, and the run time increased accordingly (19 min). Meanwhile, increasing temperature up to 40 °C, the retention time decreased, improving the resolution and separation efficiency of the studied mixture as provided by increased N values (Fig. S4); further, an increase in temperature up to 45 °C slightly decreases in N and R_s. A temperature of 40 °C was optimally selected to improve resolution and lower the analysis time.

##### Flow rate

In order to investigate its influence on the separation of the provided mixture, the flow rate was altered within a range of 0.5–2.0 mL/min. A flow rate of 2.0 mL/min was ideal to separate the studied drugs in good separation time.

### Analytical method validation

Full validation terms as indicated by ICH Q2(R1) guidelines^45^, were carried out as mentioned below (Table 2).

**Table 2:** Performance data for the determination of FF, TIO and CIC by the proposed IPC method.

Parameter	FF	TIO	CIC
Concentration range (μg/mL)	0.3–9.0	0.45–13.5	10.0–300.0
Correlation coefficient	0.9999	0.9999	0.9999
Slope	7.49	3.27	1.24
Intercept	6.72	6.54	28.03
LOD (μg/mL)	0.055	0.08	1.375
LOQ (μg/mL)	0.165	0.243	4.167
S_y/x_	0.206	0.133	0.858
S_a_	0.124	0.079	0.516
S_b_	0.027	0.01	0.003
% RSD	0.556	0.418	0.484
% Er	0.227	0.158	0.198

#### Linearity

The provided IPC method was linear over the linearity ranges provided in Table 2. Data analysis of the regression lines of the mixture was performed by giving the following equations (Table 2)

$${\text{PA}} = 6.72 + 7.49\,{\text{C}}\left( {{\text{r}} = 0.9999} \right)\quad {\text{for}}\;{\text{FF}}$$

$${\text{PA}} = 6.54 + 3.27\;{\text{C}}\left( {{\text{r}} = 0.9999} \right)\quad {\text{for}}\;{\text{TIO}}$$

$${\text{PA}} = 28.03 + 1.24\;{\text{C}}\left( {{\text{r}} = 0.9999} \right)\quad {\text{for}}\;{\text{CIC}}$$

where PA is the ratio of Peak area, C is the concentration of the medication (μg/mL) and r is the coefficient correlation (Table 2).

#### Quantification limit (LOQ) and detection limit (LOD)

USP guidelines recommended the following equations to calculate LOQ and LOD

$${\text{LOQ}} = 10\,S_{a} /b\;{\text{and}}\;{\text{LOD}} = 3.3\,S_{a} /b$$

where *b* = the slope of the calibration curve and *S*_*a*_ = the standard deviation of the intercept. LOQ and LOD values for FF, TIO, and CIC by the proposed IPC method were calculated by these equations and presented in Table 2.

#### Precision

To verify the precision and accuracy of the proposed method, the triplicate determination of FF, TIO, and CIC in two separate synthetic mixtures within the same day as intraday accuracy and for three consecutive days as in interday accuracy was carried out at three different concentrations as shown in Table 3. The small values of SD and RSD confirmed the high precision of the proposed IPC method in addition to small values of % Er revealed that revealed its accuracy.

**Table 3:** Accuracy and precision data for the determination of FF, TIO and CIC by the proposed IPC method.

	FF Conc. (μg/mL)			TIO Conc. (μg/mL)			CIC Conc. (μg/mL)		
	0.75	1.5	2.25	2.25	4.5	6.75	50.0	100.0	150.0
**Intra-day**									
${\overline{\text{X}}}$	99.11	100.12	99.74	99.82	100.30	99.31	100.33	100.16	99.71
± SD	0.2	0.79	0.36	1.19	0.98	0.93	0.85	1.16	0.74
% RSD	0.2	0.79	0.36	1.19	0.97	0.94	0.84	1.16	0.75
% Error	0.12	0.46	0.21	0.69	0.56	0.54	0.49	0.67	0.43
**Inter-day**									
${\overline{\text{X}}}$	99.23	100.36	99.67	99.64	100.54	99.37	100.10	99.30	100.13
± SD	0.22	0.51	0.52	1.29	0.64	1.05	1.17	0.68	0.23
% RSD	0.22	0.51	0.53	1.29	0.64	1.06	1.17	0.69	0.23
% Er	0.13	0.29	0.30	0.75	0.37	0.61	0.68	0.40	0.13

Each result is the mean recovery of three separate determination.

#### Accuracy

Student's t-test and variance ratio *F*-test^46^ were utilized to compare the results of the assay of FF, TIO, and CIC in MDI by applying the IPC method and the comparison HPLC method for the studied mixture. The outcomes in Tables S1 and S2 showed no significant variance between the mentioned and comparison analytical methods regarding precision and accuracy. The reference method^31^ relies on the assay of FF, TIO, and CIC applying a gradient run of RP- HPLC carried on a C_18_ column and the mobile phase composed of different ratios of acetonitrile: anhydrous disodium hydrogen phosphate buffer within 22 min. The pH was adjusted to 3.5, separation was carried out at 40° C, the flow rate of 1.0 mL/min, and UV detection at 230 nm.

#### Robustness

The stability of the peak area with deliberate minor changes in the experimental factors was evaluated using the suggested IPC method for the given mixture. These considerations include: the proportion of acetonitrile: deionized water mobile phase (55: 45 ± 2% v/v), pH (3.0 ± 0.1), and SDS concentration (0.025 ± 0.002%). This deliberate alteration did not affect the peak area of both mixtures.

#### Selectivity and specificity

Examining the impact of added substances (lactose monohydrate) displayed in MDI forms was applied to assure the selectivity of the method. The peak of the "placebo" has zero reading at the chosen wavelength of 237 nm confirming no influence from the MDI additives. Since the inhaler propellant hydrofluoroalkane, which was expelled during therapeutic actuation, had little effect on the measurements of the tested drugs in the metered-dose inhaler, the suggested methodology indicated high specificity.

#### Solution stability and mobile phase stability

No major change was observed in the response of the standard stock solution compared to the freshly prepared standard of FF, TIO, and CIC. Similarly, the stability of the mobile process was checked. In both cases, the results proved the stability of the mobile phase and the sample solution for up to seven days.

#### System suitability

USP guidelines^44^ suggest that SST parameter calculations be performed in terms of column performance (number of theoretical plates, N), resolution factor (R_s), and selectivity factor (*α*). During the process of development and enhancement, an evaluation of SST variables was performed to ensure the effectiveness of the functioning system (Table 1).

### Analysis of FF, TIO, and CIC in synthetic mixtures and combined metered-dose inhalers

Assay of FF, TIO, and CIC in their prepared mixture (Table S1) and pharmaceutical MDI (Table S2) utilizing their recommended clinical dosages 1: 1.5: 33.3, respectively were applied by the suggested approach. The accurate and precise results obtained as shown by high values of percent recovery enable quantitative assay of the studied drug mixture in quality control labs (Fig. S1).

## Conclusion

The proposed method applies the first isocratic, precise, and efficient ion-pairing liquid chromatographic (IPC) method that successfully applied for the simultaneous determination of FF (LABA), TIO (LAMA) and CIC (ICS) in their synthetic mixtures and novel combined metered dose inhaler that is widely used for the symptomatic treatment of patients with the acute chronic obstructive disease (COPD) and play a vital role in their protection against COVID-19. The provided method provided an acceptable resolution and a reasonable run time (less than 15 min). The proposed method is succussed for the separation of this ternary mixture, especially with their challenging pharmaceuticals ratio (FF, TIO, and CIC); (1: 1.5: 33.3) enabling its application in quality control laboratory.

## Supplementary Information

Supplementary Information.

## Data Availability

The datasets used and/or analyzed during the current study are available from the corresponding author upon reasonable request.
