# Supplementary material for: Isocratic ion pair chromatography for estimation of novel combined inhalation therapy that blocks coronavirus replication in chronic asthmatic patients
Source: Sci Rep. 2023 Jan 6;13:305. doi: 10.1038/s41598-023-27572-w (PMC9818053; doi:10.1038/s41598-023-27572-w)
Supplement: Supplementary file 1 — Supplementary Information. [file 41598_2023_27572_MOESM1_ESM.docx]

**
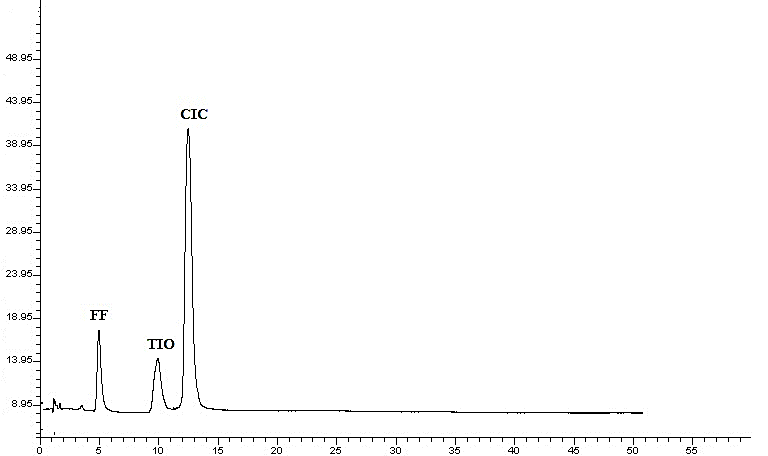
**

**Figure S1.** Typical chromatogram for the separation of FF (0.6 µg/mL, 5.8 min), TIO (0.9 µg /mL, 10.5 min.) and CIC (20 μg/mL, 13.3 min) in metered dose inhaler using ion pair mobile phase of the specified optimum characteristics.


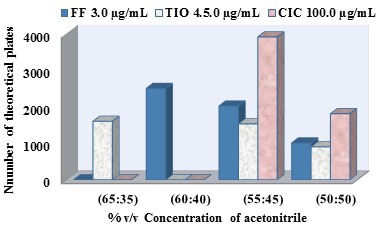


**Figure S2.** Effect of different % v/v concentration of acetonitrile: acidified deionized water on the number of theoretical plates of FF 3.0 µg/mL, TIO 4.5 µg/mL and CIC 100.0 µg/mL using mobile phase containing 0.025 % SDS pH 3.0. Flow rate, 2.0 mL/min, column temperature 40^ο^C and UV detection at 237 nm


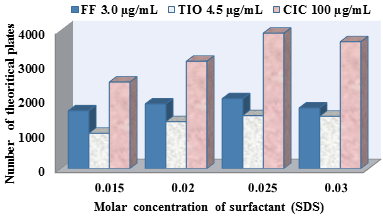


**Figure S3.** Effect of different molar concentration of surfactant (SDS) on the number of theoretical plates of FF 3.0 µg/mL, TIO 4.5 µg/mL and CIC 100.0 µg/mL using mobile phase consisting of acetonitrile: acidified deionized water pH 3.0 (55: 45% v/v). Flow rate, 2.0 mL/min, column temperature, 40^ο^C and UV detection at 237 nm.

**
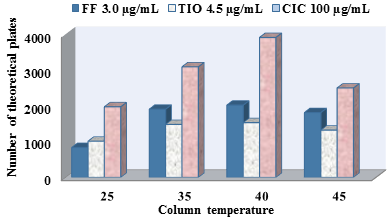
**

**Figure S4.** Effect of column temperature on the number of theoretical plates of FF 3.0 µg/mL, TIO 4.5 µg/mL and CIC 100.0 µg/mL using mobile phase consisting of acetonitrile: acidified deionized water containing 0.025% SDS (55: 45% v/v) pH 3.0. Flow rate, 2.0 mL/min, column temperature, 40^ο^C and UV detection at 237 nm.

**Table S1. Assay results for the determination of the of the FF, TIO and CIC by the proposed** **IPC method**

|  | **Proposed IPC method** | | | | | | **Reference method** **(26)** | | | | | | | | |
| --- | --- | --- | --- | --- | --- | --- | --- | --- | --- | --- | --- | --- | --- | --- | --- |
|  | **Conc. taken (μg/mL)** | | | **% found*** | | | **Conc. taken (μg/mL)** | | | **% found*** | | | | | |
|  | **FF** | **TIO** | **CIC** | **FF** | **TIO** | **CIC** | **FF** | **TIO** | **CIC** | **FF** | **TIO** | **CIC** | | |  |
| **Data** | 2.4 | 3.6 | 80 | 99.58 | 100.47 | 100.25 | 4.8 | 7.2 | 96 | 99.10 | 99.14 | | | 101.12 |  |
|  | 3.0 | 4.5 | 100 | 100.24 | 99.24 | 100.68 | 6.0 | 9.0 | 120 | 100.07 | 99.88 | | | 99.87 |  |
|  | 3.6 | 5.4 | 120 | 99.54 | 101.05 | 99.54 | 7.2 | 10.8 | 144 | 100.24 | 101.52 | | | 100.45 |  |
| **$\bar{\text{X}}$** |  | | | 99.79 | 100.25 | 100.16 |  | | | 99.82 | 100.2 | | 100.48 | | |
| **± SD** |  |  |  | 0.39 | 0.92 | 0.58 |  |  |  | 0.62 | .22 | 0.63 | | | |
| **t- value** |  |  |  | 0.04 (2.77) | 0.08  (2.77) | 0.66  (2.77) |  |  |  |  | | | | | |
| ***F-*value** |  |  |  | 2.45 (19.0) | 1.74  (19.0) | 1.18  (19.0) |  |  |  |  |  |  |  |  |  |

**^(*)^** Each result is the mean recovery of three separate determinations.

Figures between brackets are the tabulated *t* and *F*-values at (p= 0.05) (32).

**Table S2. Assay results for the determination of the FF, TIO and CIC in their combined metered dose inhaler (Triohale inhaler) by the proposed** **IPC method**

|  | **Proposed IPC method** | | | | | | **Reference method** **(26)** | | | | | | | |
| --- | --- | --- | --- | --- | --- | --- | --- | --- | --- | --- | --- | --- | --- | --- |
|  | **Conc. taken (μg/mL)** | | | **% found*** | | | **Conc. taken (μg/mL)** | | | **% found*** | | | | |
|  | **FF** | **TIO** | **CIC** | **FF** | **TIO** | **CIC** | **FF** | **TIO** | **CIC** | **FF** | **TIO** | **CIC** | |  |
| **Data** | 2.4 | 3.6 | 80 | 98.88 | 99.17 | 99.54 | 4.8 | 7.2 | 96 | 98.97 | 99.02 | | 100.26 |  |
|  | 3.0 | 4.5 | 100 | 99.37 | 98.54 | 99.81 | 6.0 | 9.0 | 120 | 99.84 | 99.73 | | 99.51 |  |
|  | 3.6 | 5.4 | 120 | 99.73 | 99.74 | 100.34 | 7.2 | 10.8 | 144 | 100.24 | 100.84 | | 100.34 |  |
| **$\bar{\text{X}}$** |  | | | 99.33 | 99.15 | 99.90 |  | | | 99.68 | 99.86 | 100.04 | |  |
| **± SD** |  |  |  | 0.43 | 0.60 | 0.41 |  |  |  | 0.65 | 0.92 | 0.46 | |  |
| **t-value** |  |  |  | 0.80 (2.77) | 1.13  (2.77) | 0.40  (2.77) |  |  |  |  | | | |  |
| ***F-*value** |  |  |  | 2.32 (19.0) | 2.34  (19.0) | 1.27  (19.0) |  |  |  |  |  |  |  |  |

**^(*)^** Each result is the mean recovery of three separate determinations.

Figures between brackets are the tabulated *t* and *F*-values at (p= 0.05) (32)
